# Evaluation and Optimization of Poly-d-Lysine as a Non-Natural Cationic Polypeptide for Gene Transfer in Neuroblastoma Cells

**DOI:** 10.3390/nano11071756

**Published:** 2021-07-05

**Authors:** Miguel Sanchez-Martos, Gema Martinez-Navarrete, Adela Bernabeu-Zornoza, Lawrence Humphreys, Eduardo Fernandez

**Affiliations:** 1 Neuroprothesis and Neuroengineering Research Group, Miguel Hernández University, 03201 Elche, Spain; miguel.sanchez17@alu.umh.es (M.S.-M.); gema.martinezn@umh.es (G.M.-N.); adela.bernabeu@gmail.com (A.B.-Z.); lawrencehumphreys@hotmail.com (L.H.); 2 Biomedical Research Networking Center in Bioengineering, Biomaterials and Nanomedicine (CIBER-BBN), 28029 Madrid, Spain

**Keywords:** neuroblastoma, SH-SY5Y, HeLa, 3T3, PDL, CPP, gene therapy, nonviral vector poly-lysine

## Abstract

Cationic polypeptides and cationic polymers have cell-penetrating capacities and have been used in gene transfer studies. In this study, we investigate the capability of a polymer of d-lysine (PDL), a chiral form of α–Poly-lysine, as a possible nonviral vector for releasing genetic materials to neuroblastoma cells and evaluate its stability against proteases. We tested and compared its transfection effectiveness in vitro as a vehicle for the EGFP plasmid DNA (pDNA) reporter in the SH-SY5Y human neuroblastoma, HeLa, and 3T3 cell lines. Using fluorescent microscopy and flow cytometry, we demonstrated high transfection efficiencies based on EGFP fluorescence in SH-SY5Y cells, compared with HeLa and 3T3. Our results reveal PDL as an efficient vector for gene delivery specifically in the SH-SY5Y cell line and suggest that PDL can be used as a synthetic cell-penetrating polypeptide for gene therapy in neuroblastoma cells.

## 1. Introduction

Gene therapy is a technique that alters gene expression to change the biological properties of cells for therapeutic purposes [1] and has proven to be a promising tool. There are several methods to achieve this objective, some of which include changing pathologic genes for functional genetic sequences, inactivating a defective gene, or treating a pathological condition by inserting a new gene [1]; however, many challenges remain [2,3]. One of the main problems is the difficulty in delivering the nucleic acids through the cell membrane. The most common carriers are viral vectors [4], but some studies have demonstrated that viruses have several limitations related to immunogenicity, the size of the nucleic acids they can transport, and the risk of mutagenesis [5]. In this framework, there is a clear need for the development of new approaches in gene therapy [6,7] and to look for alternative tools to viruses, known as nonviral vectors, among which are cationic polypeptides and cationic polymers.

Cell-penetrating peptides (CPPs) are small peptides with between 7 to 30 residues of amino acid size, which exhibit improved cellular penetration through the plasma membrane [8]. CPPs can form complexes and carry peptides, drugs, nucleotide chains, enzymes, etc. Several CPPs have a biological origin such as the TAT protein [9] or Antp [10] and can also be produced synthetically [11]. CPPs can be linked to other molecules for transport through disulfide linkage, thioester linkage, or by creating chimeric proteins with other CPPs or proteins. The noncovalent formulation involves electrostatic or hydrophobic interactions between the molecule and the CPP [12]. 

The mechanisms by which CPP’s penetrate through cell membranes can be by two distinct pathways—energy-dependent internalization by endocytosis mechanism or direct translocation through the cell membrane [8]. In addition, CPPs can be classified into three categories: peptide-based proteins such as TAT and penetratine, chimeric peptides such as transportan, and synthetic-based peptides such as polylysine (PLL) [13]. Moreover, CPPs can be further classified according to their physical–chemical properties as follows: cationic peptides, which are those that are made up of short sequences of amino acids that are mainly arginine, lysine, and histidine (TAT and PLL); amphipathic peptides, which are those with a polar domain and a nonpolar domain (MPG); hydrophobic peptides, which are formed by nonpolar residues of valine, leucine, and tryptophan [8,14].

CPPs have been used as nonviral vectors to introduce diverse compounds into cells for biomedical applications, such as drugs [15], proteins [16], quantum dots [17], radiolabeled antibodies [18], and nucleic acids such as siRNA or DNA [19]. The TAT peptide was one of the first CPPs used for gene delivery, due to its cationic nature and the abundance of arginine and lysine residues, which have a positive charge that interacts with the negative charges of nucleic acids [20]. Until now, more than 1700 peptide sequences of both biological or synthetic origin have been described and classified as CPPs [21]. As nonviral vectors, CPPs have been used to treat pathologies, such as lung diseases, with an octo–arginine conjugate to an amphiphilic region to improve DNA condensation, followed by HS-GAG binding domain to enhance transduction, and finally, a polietilenglicol chain to protect therapeutic pDNA from the action of nucleases [22]. An example of a synthetic custom-designed CPPs are the POD “peptide for ocular delivery,” a GGG(ARKKAAKA)4 chain used for drug delivery treatment related to eye diseases. Here, they showed that POD is capable of condensing siRNA and pDNA and can also pass through the cell membrane of the neural retina, photoreceptors, ganglion cells, as well as enter in the sclera, choroids, and into the dura of the optic nerve via topical application [23].

Poly-l-lysine (PLL) was one of the first synthetic polypeptides that were studied for its DNA condensation capacity [24]. Its ability to transport genetic material inside certain types of cells was tested in vitro and in vivo [25]. However, PLL is usually joined to other proteins and synthetic compounds such as polyethylenimine (PEI) when used as a vector and unconjugated polylysines have low transfection rates [26]. Furthermore, PLL exhibits positive electrostatic charges at a high density, which can induce some side effects such as cytotoxicity and membrane disruption [27,28]. 

A possible alternative to PLL is to use the poly-d-lysine (PDL), a chiral form of α–polylysine, which is widely used to improve cell adherence in neuronal cultures [29] and is relatively inexpensive and commercially available. PDL has been used in transfection assays conjugated to the cholera toxin B chain [30,31], and with the RAP protein to target human hepatoma cells [32]. Chirality and transfection capacity of PDL in HeLa cells has also been reported [33]. Moreover, its use has also been described in the systemic delivery of a PDL–plasmid complex in vivo [31] and in clinical trials to treat cystic fibrosis using conjugated PDL–peg [34]. In this context, PDL induces a low immune response [35] and can be used to increase cell-type specificity [36]. 

Here, we assess the potential of long-term PDL application for gene transfer in SH-SY5Y human neuroblastoma and compared its capacity to transfect the enhanced green fluorescent protein plasmid (EGFP) in HeLa and 3T3 cells. Furthermore, we evaluated its stability against proteases as its effects on cell viability. We selected neuroblastoma cells since this cancer is the most common malignant tumor found in infancy [37,38] and has a survival rate of less than 20% [39]. Neuroblastoma treatments are based mainly on surgery, chemotherapy, and radiotherapy, but because of the nature of the disease, it can metastasize into other tissues with ease [38]; therefore, recovery rates are generally low [40]. Furthermore, frequent complications of these treatments include high levels of drug toxicity and low specificity on the target tissue, as well as the adaptability of the tumor cells to the drugs used, leading to what is known as refractory cancer [41]. Consequently, it is imperative to discover alternative treatments, and gene therapy has been proposed as a potential tool for treating this tumor [39]. We tested several DNA–PDL complexes and show that PDL has a high transfection efficiency in the SH-SY5Y neuroblastoma cell line when compared to HeLa and 3T3 cell lines. These results suggest that PDL can be used as a synthetic cell-penetrating polypeptide for gene therapy in neuroblastoma cells [42]. 

## 2. Materials and Methods 

### 2.1. Ethical Approval

All experimental procedures conformed to directive 2010/63/ EU of the European Parliament and Council and the RD 53/2013 Spanish regulation on the protection of animal use for scientific purposes and approved by the Miguel Hernandez University Committee for Animal use in Laboratory.

### 2.2. Plasmid/PDL Interaction Assay

The pCMS-EGFP plasmid (5500 bp) bought from Addgene (Watertown, MA, USA) was provided by the Clontech laboratory PT3268-5, and the coding for the enhanced green fluorescent protein (EGFP) was stored at −80°. It was amplified in our facilities with *E. coli* bacteria and a QIAfilter plasmid kit provided by Qiagen at a final concentration of 1 mg/mL. PDL solution was prepared at 1 mg/mL 70/150 kDa (Thermo Fisher Scientific, Waltham, MA, USA). The cell lines SH-SY5Y, HeLa, and 3T3 (Sigma-Aldrich, Inc., St. Louis, MO, USA) were placed in an Eppendorf with heat-inactivated fetal bovine serum (FBS) (Biowest, Riverside, MO, USA) and 10% of dimethyl sulfoxide (DMSO) (Merk, Darmstadt, Germany) and stored at −80 °C until used.

To check for interactions between lysines and the plasmid, we incubated PDL (1 mg/mL) and EGFP plasmid (1 mg/mL) for an hour. PDL concentrations were varied to obtain several plasmids to PDL ratios (*w*/*w*). Interactions were analyzed by electrophoresis running the samples on an agarose gel at a 0.8% concentration. GreenSafe (NZYTech, Lisboa, Portugal) was used as a DNA stain. The mix was left to run for 45 min at room temperature at which point the gel was illuminated with a UV lamp, and the images were taken with a transilluminator (Vilber Luomart, Marne-la-Vallée, France).

### 2.3. DNase I Protection Assay

To analyze PDL’s ability to protect the pDNA from enzymatic degradation, we carried out a DNase I (Sigma-Aldrich, Darmstadt, Germany) protection assay. pDNA–PDL complexes were prepared at *w*/*w* ratios of 1:2, 1:4, and control naked pDNA was incubated at 37 °C for 60 min with DNase I (1 U/μg of DNA), 1 μL MgCl_2_ 25 mM (Sigma-Aldrich, Darmstadt, Germany), at which point 1 μL EDTA 50 mM (Sigma-Aldrich, Darmstadt, Germany) was added and samples were heated at 65 °C for 10 min to inactivates DNase I. Heparin (500 U/μg of DNA) (Hospira-Pfizer, New York, NY, USA) was added for 1 h to release DNA from the PDL. The samples were analyzed by way of 0.8% agarose gel electrophoresis. GreenSafe was used to dye the pDNA. The complexes were left to run for 45 min at 90 V at room temperature before the gel was exposed to a UV light, and the images were acquired using a transilluminator (Vilber Luomart, Marne-la-Vallée, France). 

### 2.4. Elaboration of DNA–Poly-d-Lysine Complex

The DNA–PDL complexes were made by mixing EGFP plasmid with different volumes of PDL to obtain different plasmid/PDL ratios ranging from 1:0.12 to 1:4. For the positive control, we used a mix of DNA/Lipofectamine (1:1) (Thermofisher, Darmstadt, Germany) whereas for negative controls we used the naked plasmid. All compound mixtures were prepared at a final volume of 100 µL mixed with distilled water and were incubated at room temperature for 30 min to allow the interaction between the plasmid and PDL. 

### 2.5. MTT Assay

Mitochondrial respiration was used as an indicator of cell viability because of its ability to convert Thiazolyl Blue Tetrazolium Bromide (MTT) (Sigma-Aldrich, Darmstadt, Germany) into formazan. Culture cell viability was performed using the MTT assay in 96-well plates. SH-SY5Y, HeLa, and 3T3 cells were seeded at 2 × 10^2^ cells per well in 100 µL of culture medium and grown for 48 h at 5% CO_2_ and 37 °C room temperature. After this time, we carried out the first treatment with our 1:2 or 1:4 plasmid/PDL (80 ng of pDNA per well) mixture and left it in the culture medium for 48 h. We then replaced the entire culture medium and added fresh plasmid/PDL mixture and repeated the same process twice more every 48 h. Thus, cells were incubated in either the plasmid/PDL mixture ratio of 1:2 or 1:4 for either 48, 96, or 144 h for comparative testing. All cells irrespective of treatment duration were left to grow for 9 days at which point they were analyzed. Culture medium was completely exchanged with fresh culture medium with the addition of MTT at 1 mg/mL concentration and was left to incubate for 4 h. The culture medium was then removed, and 100 µL/well of dimethyl sulfoxide DMSO (Sigma-Aldrich, Darmstadt, Germany) was added to dissolve formazan crystals. Absorbance was measured at 595 nm in a microplate reader. Comparisons were performed using untreated cells as the control and normalized to 100% viability as the reference. 

### 2.6. Assays In Vitro of SH-SY5Y Cells

The SH-SY5Y human neuroblastoma, HeLa, and 3T3 cell lines were seeded on 24-well plates with a 12 mm diameter coverslip, which was pretreated with PDL (0.0125 mg/mL) and laminin (0.0083 mg/mL) (Sigma-Aldrich, Darmstadt, Germany) at a concentration of 1 × 10^3^ per well in 500 µL of culture medium and left to grow in an incubator (37 °C and 5% CO_2_) for 72 h. Cells were then treated with varying plasmid/PDL ratios for a duration of different times, as previously described above (400 ng of pDNA per well). This is depicted in Table 1. Control cells were transfected with EGFP/lipofectamine two days after seeding for a total of 144 h with medium and EGFP/lipofectamine (1:1) replenished every 48 h. 

Cells were then fixed with 500 µL of paraformaldehyde (PFA) 4% (v/p) (Sigma-Aldrich, Darmstadt, Germany) for 5 min in the same plate in which the cells were cultured. PFA was removed and another 500 µL of PFA 4% was added again for 15 min at which point it was removed and cells were washed, once with 500 µL phosphate buffer solution (PBS) (Sigma-Aldrich, Darmstadt, Germany) and 1 µL Hoechst (1 µg/µL) per well for 5 min and washed twice more with PBS and kept at 4 °C.

### 2.7. Cell Count Assay

Samples were analyzed on a fluorescence microscope with a 488 nm filter for EGFP expression in transfected cells and a 358 nm filter for Hoechst-stained nuclei. Images were captured with a Zeiss Apotome 2.0 fluorescent microscope with a 20× objective. In total, 10 areas were randomly selected, each containing a minimum of 500 nuclei. Cells were manually counted in a blind manner. 

### 2.8. Flow Cytometer Assay

In a 24-well plate previously treated with PDL (0.0125 mg/mL) and laminin (0.0083 mg/mL), 1 × 10^3^ cells were seeded directly over the well using the same protocol and the transfection process that was described in the previous section. On the ninth day after the seeding, cells were washed with PBS at a temperature of 37 °C and were treated with 200 µL 0.05% Trypsin–EDTA (1×) (Gibco, Darmstadt, Germany, 64293) for 5 min in a 37 °C incubator to detach the cells from their support. Then, 400 µL of cold phosphate buffer and 20% FBS were added to block trypsinization. Cells were then placed in a 1.5 mL Eppendorf and centrifuged at 200 g’s for 5 min. After that, the supernatant was removed and the pellet was resuspended in 1 mL of PBS +2% FBS, and samples were conserved on ice. Transfected cells expressing EGFP were measured with a flow cytometer (SONY SH800) at Ex/Em = 488/510 nm wavelength. Over 10,000 counts were made in each of the three experiments that we performed, the data were processed with Flowing Software (version 2.5.1, Turku Centre for Biotechnology University of Turku, Finland, 2013). Cell expression data were expressed as the percentage of positive fluorescent cells. As a negative control, we compared cells that had been treated with naked DNA. 

### 2.9. Statistical Analysis

Data are represented as mean ± SD. Statistical analysis was made using a two-sample Student’s t-tests. Each experiment was repeated at least three times.

## 3. Results

### 3.1. Gel Retardation Assay

**Figure 1:**
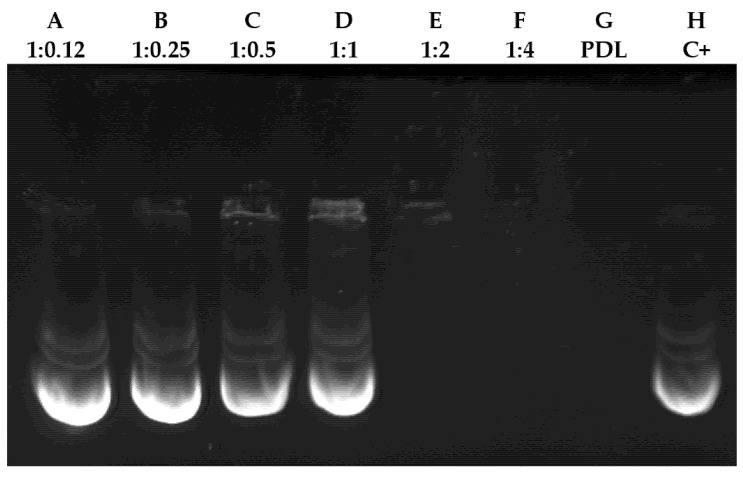
Interaction analysis between different plasmid/PDL ratios. Interaction analysis of plasmid–PDL complex at different ratios in agarose gel (0.8%) stained with GreenSafe. Columns A–F show the different plasmid/PDL ratios used (1:012, 1:0.25, 1:05, 1:1, 1:2, 1:4 and 1:8 respectively). Column G only contains PDL and column H was used as a positive control where only DNA was inserted.

Interactions between the plasmid–PDL complexes were evaluated by electrophoretic mobility shift assays using incremental ratios (Figure 1). The mobility of ratios ranging from 1:0.12 to 1:1 (lanes A–D) was comparable to that of naked DNA (Figure 1H). However, we observed that plasmid/PDL ratios of 1:2 to 1:4 interact sufficiently with each other to form a complex that is able to block the migration of DNA through the gel. 

### 3.2. DNase I Protection and Release Assay

**Figure 2:**
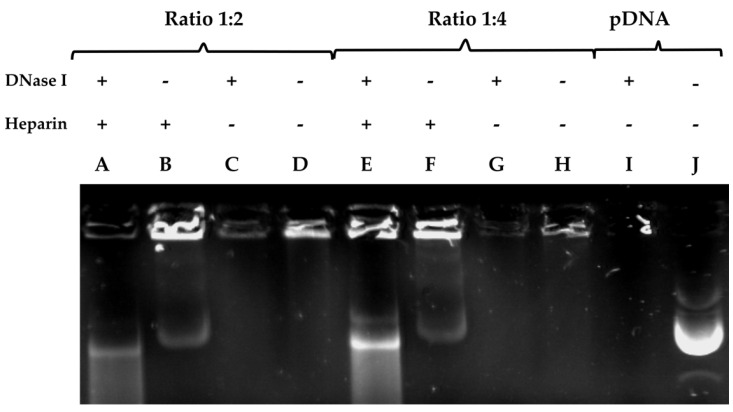
pDNA liberation assay from varying plasmid/PDL ratios using heparin. DNA complexes were formed with PDL at different ratios and run on a 0.8% agarose gel electrophoresis stained with GreenSafe. The protective properties of PDL were assessed after heparin released the DNA from the complex and DNase 1 was then allowed to cleave it. In columns A–D a plasmid-DNA ratio of 1:2 was inserted. Column A was run with the addition of DNase 1 and heparin. Column B only contained heparin and column C only contained DNase 1. In column D only the plasmid-DNA complex was inserted. Columns E–H were run with a plasmid-DNA ratio of 1:4. Column E was run with the addition of DNase 1 and heparin. Column F only contained heparin and column G only contained DNase 1. Column I only contained the plasmid-DNA complex. Column J was a positive control whereas only the naked DNA was inserted. + or − symbols indicate the presence or absence of heparin or DNase I.

One of the essential properties that all vectors should exhibit for any in vivo experiments is the protection of the DNA they package and transport from the nucleases present in the blood and tissue [43]. In the DNase protection and release assay (Figure 2), we evaluated the capacity of PDL at a ratio of 1:2 or 1:4 to protect the plasmid DNA from digestion from DNase I. Heparin was used to liberate the DNA from the plasmid–PDL complexes. For both ratios, we observed that PDL was able to form a complex with the plasmid and partially protect it from being digested by DNase I, which was partly liberated by heparin (lane A and E). When we added heparin without DNase I, we observed the partial liberation of the DNA from the PDL complex (lane B and F). When we added DNase I without heparin, the DNA was not liberated (lane C and G). Similar results were observed when neither was added (lane D and H), and no band was formed. When using a ratio of 1:4, we observed a more pronounced liberation of DNA, in comparison to a 1:2 (lane A and E). In lane I, we added DNase I to naked plasmid DNA where no band was observed, and in lane J, we added DNA for comparative controls. It is possible that in the loading sites (B, D, E, F, H), PDL interferes in the binding between the plasmid and the GreenSafe DNA stain, and that heparin can liberate the plasmid from the PDL complex, allowing for the DNA to be stained. However, more experiments are still needed for more definitive conclusions to be drawn. 

### 3.3. Cell Viability Evaluated according to PDL Concentration and Duration of Administration

**Figure 3:**
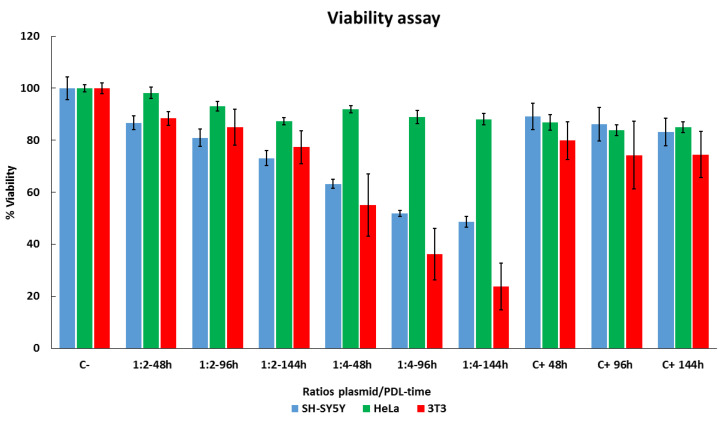
Evaluating cell viability with long-term DNA–PDL treatments in SH-SY5Y, HeLa, and 3T3 cells. Cells were treated with two ratios of plasmid/PDL 1:2 or 1:4 two days after seeding for 48 h, 96 h, and 144 h, and compared with control untreated cells 9 days after seeding. Cellular viability was measured using an MTT assay. Control cells were referenced as 100% viability for comparisons. These experiments were repeated three times and data are shown as mean ± standard deviation (SD).

To evaluate the effects of PDL on cell survival, we used an MTT assay to test viability. We tested different DNA/PDL ratios and varied the time SH-SY5Y, HeLa, and 3T3 cells were exposed to the complex. Two days after seeding the cells, all the media in each well was completely replaced. DNA–PDL complex was then applied and left to mix with the cells for 48 h. This process of medium change and a new addition of DNA–PDL complex was repeated every 48 h, i.e., at 96 h and 144 h. Nontreated cells were used as a control and compared as a 100% reference. Results are shown in Figure 3. HeLa cells exhibited the highest viability throughout all treatments, in comparison to SH-SY5Y and 3T3 cells. Specifically, using a ratio of 1:2 for 48 h, we observed the highest viability in HeLa cells (98 ± 2.2% *p* = 3.0 × 10^−1^), followed by 3T3 cells (88.39 ± 2.67% (*p* = 1 × 10^−3^)), and then by SH-SY5Y cells (86.7 ± 2.72% (*p* = 9 × 10^−3^)). A similar trend was observed at 96 h, although viability decreased in all three cell lines (HeLa = 92.98 ± 1.82% (*p* = 2 × 10^−4^), 3T3 = 85 ± 6.9% (*p* = 2 × 10^−3^), SH-SY5Y = 80.88 ± 3.3% (*p* = 2 × 10^−3^)) and at 144 h (HeLa = 87.28 ± 1.36% (*p* = 6.43 × 10^−5^), 3T3 = 77.31 ± 6.23% (*p* = 2.1 × 10^−4^), SH-SY5Y = 73.15 ± 2.86% (*p* = 5.85 × 10^−5^)). Using a ratio of 1:4 decreased viability in all three cell lines when compared to 1:2 with HeLa cells, once again exhibiting the highest viability. At 48 h, a 1:4 treatment resulted in the highest viability in all three cell lines when compared to negative controls (HeLa = 91.97 ± 1.4% (*p* = 3.54 × 10^−5^), 3T3 = 55 ± 11.89% (*p* = 2.24 × 10^−5^), SH-SY5Y = 63.21 ± 2.86% (*p* = 2.45 × 10^−5^)). At 96 h, we observed a further decrease in viability in all three cell lines (HeLa = 88.93 ± 2.64% (*p* = 1.06 × 10^−4^), 3T3 = 36.15 ± 9.9% (*p* = 1.36 × 10^−7^), SH-SY5Y = 51.91 ± 1.19% (*p* = 2.12 × 10^−6^)), which was even more pronounced at 144 h (HeLa = 88.11 ± 2.24% (*p* = 7.43 × 10^−5^), 3T3 = 23.76 ± 8.99% (*p* = 2.1 × 10^−3^), SH-SY5Y = 48.61 ± 2% (*p* = 9.09 × 10^−7^)). 

### 3.4. Cell-Type-Specific Differences in EGFP Expression Using PDL 

**Figure 4:**
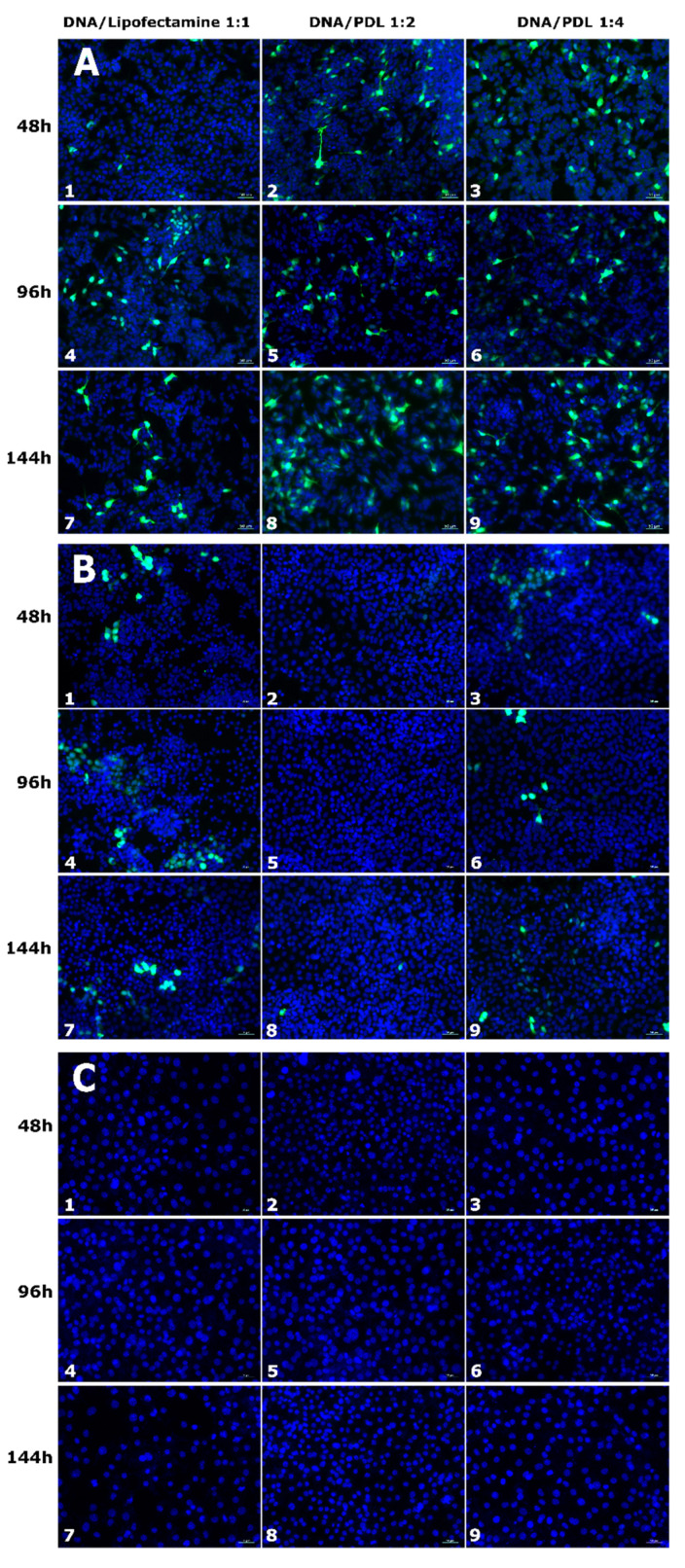
EGFP expression in SH-SY5Y (**A**), HeLa (**B**), and 3T3 (**C**) cells transfected with PDL or lipofectamine. EGFP expression (green) in cells treated with lipofectamine (1, 4, 7 columns), DNA/PDL (1:2) (2, 5, 8 columns) and DNA/PDL (1:4) (3, 6, 9 columns) for 48 h (1, 2, 3 rows), 96 h (4, 5, 6 rows) and 144 h (7, 8, 9 rows) in each cell line. Cell nuclei were stained with Hoechst (blue). Scale = 50 µm.

**Figure 5:**
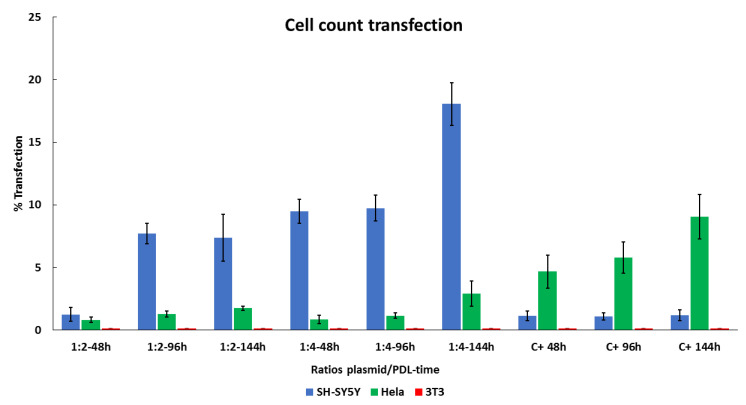
Percentage of transfection in each cell line by cell count. Results are be expressed as EGFP positive cells/total counted cells number counting at least 500 nuclei of each sample. Data are represented by mean ± SD of three distinct experiments.

We used confocal microscopy imaging to evaluate the optimal transfection protocol in terms of EGFP/PDL ratios (1:2, 1:4) and administration duration (44, 96, and 144 h) in SY5Y, HeLa, and 3T3 cells lines. Transfection efficiency for each condition was determined by comparing EGFP positive cells to lipofectamine treated control cells (1:1). Figure 4 shows representative images of EGFP expression, and the results of manual cell counts are shown in Figure 5. In general, the transfection activity of lipofectamine on SH-SY5Y after 48 h cells was very low. Using a PDL ratio of 1:2, we observed improved EGFP expression, in comparison to lipofectamine. At 48 h of treatment, a slight increase in EGFP transfection of 1.24 ± 0.56% was observed, although this was not statistically different when compared to with control cells transfected with lipofectamine treated for the same amount of time, 1.13 ± 0.37%. However, after 96 h, EGFP expression increased to 7.73 ± 0.8% (*p* = 6.45 × 10^−7^), comparative to lipofectamine treatment for the same duration, with 1.10 ± 0.28%. Similar results were obtained at 144 h of treatment with an increase of 7.37 ± 1.87% (*p* = 9.26 × 10^−4^) when compared to lipofectamine, with 1.19 ± 0.41%. Incrementing the ratio to 1:4 resulted in higher expression for all treatments. At 48 h, 96 h and 144 h we observed an 9.49 ± 0.97% (*p* = 3.8 × 10^−8^), 9.75 ± 1.02% (*p* = 8.27 × 10^−7^) and 18.06 ± 1.7% (*p* = 7.8 × 10^−8^) increase, respectively, when compared to lipofectamine. However, when transfecting HeLa Cells with our plasmid–PDL complex, we observed a decrease in EGFP expression when compared to lipofectamine irrespective of duration or ratio used (48 h lipofectamine = 4.69 ± 1.3%, PDL ratio 1:2 = 0.83 ± 0.21% (*p* = 4.0 × 10^−2^), ratio 1:4 = 0.86 ± 0.32% (*p* = 2.1 × 10^−2^)); (96 h lipofectamine = 5.78 ± 1.24%, PDL ratio 1:2 = 1.29 ± 0.26% (*p* = 2.5 × 10^−2^), ratio 1:4 = 1.16 ± 0.21% (*p* = 2.2 × 10^−2^)); (144 h lipofectamine = 9.05 ± 1.78%, PDL ratio 1:2 = 1.76 ± 0.16% (*p* = 9.6 × 10^−3^), ratio 1:4 = 2.91 ± 1.01% (*p* = 5.1 × 10^−3^)). We did not observe any EGFP expression, neither when using lipofectamine nor with our plasmid–PDL complexes at any duration of application in 3T3 cells (Figure 3C). 

**Figure 6:**
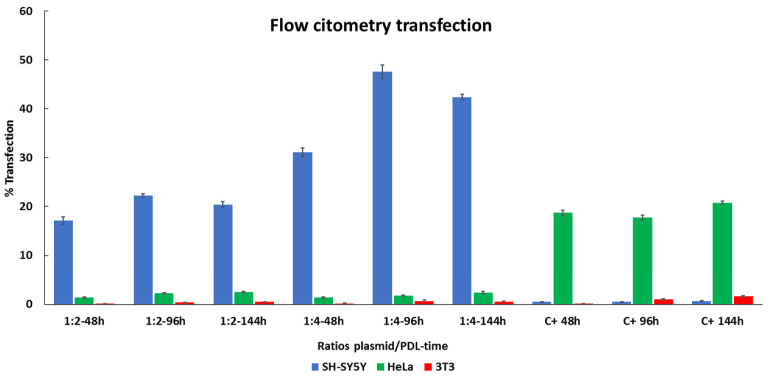
Comparing EGFP expression between PDL and lipofectamine determined by flow cytometry. SH-Sy5Y, HeLa, and 3T3 cells were treated three times (48, 96 and 144 h) with two different ratios of plasmid/PDL (ratio 1:2 and ratio 1:4) and with lipofectamine as a positive control (C+). Data are represented by mean +/− SD of three distinct experiments.

To further compare transfection efficiency in SH-SY5Y, HeLa, and 3T3 cells between PDL and lipofectamine, we used flow cytometry for the quantification of EGFP expression [44]. Figure 6 shows the quantitative results. In SH-SY5Y cell line, increased transfection levels were observed, with the ratio 1:2 for all time durations of drug application when compared to lipofectamine (lipofectamine 48 h = 0.48 ± 0.01%, 96 h = 0.5 ± 0.01%, 144 h = 0.79 ± 0.01%, PDL 48 h = 17.11 ± 0.74% (*p* = 2.5 × 10^−3^), 96 h = 22.24 ± 0.36 (*p* = 3.6 × 10^−4^), 144 h = 22.24 ± 0.36 (*p* = 3.6 × 10^−4^)). This was even more pronounced using a ratio of 1:4 at all-time durations (48 h = 31.10% (*p* = 1.2 × 10^−3^), 96 h = 47.61 ± 1.3% (*p* = 1.1 × 10^−3^), 144 h = 42.37 ± 0.54% (*p* = 2.2 × 10^−4^)). These results demonstrate a stronger expression in SH-SY5Y cells in comparison to lipofectamine and is dependent on the duration of treatment.

The highest expression obtained using lipofectamine was observed in HeLa cells, which was consistent throughout each of the time durations (48 h = 18.68 ± 0.59%, 96 h = 17.72 ± 0.59%, 144 h = 20.79 ± 0.34%). However, when comparing with PDL, the expression levels were considerably lower (48 h = 1.40 ± 0.1% (*p* = 2.8 × 10^−4^), 96 h = 2.29 ± 0.13% (*p* = 3.6 × 10^−4^), 144 h = 2.54 ± 0.1% (*p* = 4.6 × 10^−4^)). Similar results were obtained using a PDL ratio of 1:4 (48 h = 1.33 ± 0.18% (*p* = 6.2 × 10^−4^), 96 h = 1.79 ± 0.11% (*p* = 2.8 × 10^−4^), 144 h = 2.40 ± 0.24% (*p* = 9.0 × 10^−4^)). Although expression was relatively low for both 1:2 and 1:4 ratios, we did observe increments after each treatment. These results suggest that HeLa cells prefer lipofectamine over PDL in terms of transfection efficiency. 

Relatively low levels of expression were observed when transfecting 3T3 cells with lipofectamine, with slight increments after each treatment (48 h = 0.13 ± 0.005%, 96 h = 1.05 ± 0.14%, 144 h = 1.63 ± 0.11%). Using a ratio of 1:2 PDL exhibited comparatively lower expression (48 h = 0.084 ± 0.011% (*p* = 4.3 × 10^−3^), 96 h = 0.36 ± 0.03% (*p* = 3.9 × 10^−2^), 144 h = 0.49 ± 0.04% (*p* = 5.8 × 10^−3^)), in comparison to lipofectamine, although we did observe slight increments again after each treatment. A similar trend was observed when using a PDL ratio of 1:4 (48 h 0.16 ± 0.03% (ns), at 96 h, 0.67 ± 0.14 (*p* = 4.6 × 10^−2^), and at 144 h, 0.53 ± 0.03% (*p* = 6.3 × 10^−3^)). Moreover, 3T3 cells exhibit the poorest expression when using PDL to transfect comparatively to the other cells lines. All together, these results suggest that SH-SY5Y cells have a higher affinity for PDL over lipofectamine and lipofectamine appears to favor expression in other cells over SH-SY5Y cells.

## 4. Discussion

This study shows the capacity for PDL to package and protect pDNA from potential nucleases normally found in blood serum [45] and act as a preferential vehicle of genetic material to SH-SY5Y human neuroblastoma cells when compared to HeLa and 3T3 cells. Neuroblastoma is one of the most common types of tumors in childhood, with a low recovery rate and high mortality levels [37,38]. Although several approaches and strategies for treatment have been developed for this tumor, the results obtained so far have been limited, and this has prompted us to investigate alternative therapies. Gene therapy has been proposed as a potential tool for treating neuroblastoma [39], but the efficiency of generic transfection reagents in vitro such as lipofectamine and Turbofectin is generally less than 5% [46]. Our results also confirm very low transfection ratios with lipofectamine; hence, there is a clear need to develop more effective gene transfer systems for neuroblastoma cells, and PDL could open a new path in this field. 

As arginine is the amino acid residue most abundant in the TAT peptide [47], the majority of studies have focused on arginine-based CPPs as a transport vehicle for genetic material or drug molecules [47,48]. Most of the literature that describes lysine-based CPPs as transporter report some sort of linkage between another compound to the L-form. To date, even less exists in the literature in respect to the D-form, which is a commercial compound normally used to treat surfaces for cell attachment [29] as a nonviral vector for gene transfer [28]. Nonetheless, our results demonstrate that PDL can also act as a nonviral vector and that consecutive treatments are well tolerated by cells and have an additive effect for transfections. 

We confirmed that PDL can transfect the plasmid reporter EGFP with high affinity in the neuroblastoma cell line SH-SY5Y with higher efficiency when compared to HeLa and 3T3. When we compared PDL with lipofectamine, a widely used gene delivery reagent for cell transfections, we observed up to 98 times more transfection rates. This increase surpasses results also reported for TurboFectin 8.0, a preferred DNA carrier in SH-SY5Y cells [46]. This opens interesting possibilities for the exploration of different CPP D-form variants as vectors for gene therapy-based treatments or as carriers for other types of cargoes such as drugs or molecules for image diagnosis. PDL presents a possible alternative to viruses as a vector overcoming the size limitation in plasmid size incorporation they present [39]. The ratio of plasmid/PDL in which we observed expression and the lowest toxicity was 1:2. Increasing the ratio to 1:4 yielded higher expression but resulted in less viability in culture. However, further studies are necessary to check if the drop in viability we observed at ratio 1:4 may be due to cytotoxic or a cytostatic process. More efforts also need to be carried out to determine the mechanisms involved in the cell type preference for SH-SY5Y with PDL, in comparison to HeLa and 3T3 cells, and whether this specificity can be capitalized on and transferred to other cell types.

Our methodology of replacing all the old medium every 48 h with fresh medium and adding a new plasmid–PDL mix each time appears to avoid possible d-lysine toxicity. These consecutive additive transfections appear to be well tolerated by cells and exhibit more accumulative expression when compared to lipofectamine. We chose this experimental design to simulate possible long-term treatments in vivo. Most in vitro transfection times range from 1 [49] to 48 h [50], with 4 h being the most commonly used [7,51]. Our results suggest that this method of changing the medium every 2 days and reapplying fresh DNA–PDL complex could be adapted for in vivo purposes. In our, in vitro assay, we were unable to test viability past 9 days since our cell lines reached the limit of confluence. For in vivo use, a balance would need to be determined between the least toxic ratio of plasmid–PDL, which can be administered and tolerated for prolonged periods of time. We observed in vitro that repetitive doses of PDL result in increased expression and are concentration dependent. This, however, also directly influences cell viability with longer exposure to PDL at higher concentrations resulting in less viability. The preference for PDL uptake by SH-SY5Y cells also opens the possibility for further studies into its use at higher concentrations to target these cells detrimentally. 

According to size classification, peptides are defined as anything between 2 and 60 amino acids [52,53], while proteins are classed as anything larger [21], but there is not an official border between peptide and protein size. The commercially available PDL we use forms different size fragments, ranging from 70 to 150 amino acids, and therefore is classed as a protein. Hence, we cannot strictly define it as a CPP. We, therefore, suggest placing it in its own category and have coined it a cell-penetrating protein (CPPro). 

In seeking alternative transfection vectors [54], the most important features are high transduction capacity, persistent chronic transfection, low toxicity, and inexpensive production costs. Here, we demonstrate that PDL serves as an alternative nonviral vector that possesses the aforementioned requirements and can transfect mammalian cells. 

In this study, we transfected cells with varying fragment sizes of PDL (70−150 amino acids). Further investigation needs to be carried out to determine if in this size range there is a fragment size that interacts optimally with plasmids or drug compounds. Additional testing in different plasmid sizes also needs to be performed to fully evaluate its potential as an all-purpose nonviral transfecting reagent.

Building on the results, we have seen with PDLs preferential uptake with DNA in neuroblastoma cells that it would be interesting to evaluate its capacity to transport RNA through the cell membrane. This would open new possibilities to target using siRNAs certain genes related to neuroblastomas such as MYCN, which is overexpressed in more than 25% of high-risk diagnosed patients [55,56]. Other candidates for targeting include the ST8SIA1 and B4GALNT1 genes, which are directly involved in the synthesis of GD2 [57,58], a glycosylated lipid molecule belonging to the class of glycosphingolipids, which are involved in the adhesion of cancerous cells to the extracellular matrix. 

These preliminary results show that PDL is a promising candidate as a vehicle in gene therapy with preferential uptake by SH-SY5Y cells, opening further possibilities to explore its use for the treatment of human neuroblastoma. 

## Figures and Tables

**Table 1:** Preparation of 24-well plates. The numbers 1, 2, 3, and 4 are the columns in plates, and A, B, and C are the rows in the plate. Cells were fixed on the ninth day. X denotes a treatment, (-) denotes only a change in medium.

		1	2	3	4
Time		Ratio 1:2	Ratio 1:4	C+	C−
3rd day (48 h)	A	X	X	X	-
	B	X	X	X	-
	C	X	X	X	-
5th day (96 h)	A	-	-	-	-
	B	X	X	X	-
	C	X	X	X	-
7th day (144 h)	A	-	-	-	-
	B	-	-	-	-
	C	X	X	X	-

## Data Availability

Data can be available upon request from the authors.
